# Deciphering key genomic regions controlling flag leaf size in wheat via integration of meta-QTL and in silico transcriptome assessment

**DOI:** 10.1186/s12864-023-09119-5

**Published:** 2023-01-19

**Authors:** Binxue Kong, Jingfu Ma, Peipei Zhang, Tao Chen, Yuan Liu, Zhuo Che, Fahimeh Shahinnia, Delong Yang

**Affiliations:** 1 State Key Laboratory of Aridland Crop Science, Lanzhou, 730070 China; 2 College of Life Science and Technology, Gansu Agricultural University, Lanzhou, 730070 China; 3 Plant Seed Master Station of Gansu Province, Lanzhou, 730000 China; 4 Bavarian State Research Centre for Agriculture, Institute for Crop Science and Plant Breeding, 85354 Freising, Germany

**Keywords:** Wheat, Flag leaf traits, QTL mapping, Meta-analysis, Transcriptome, Candidate genes

## Abstract

**Background:**

Grain yield is a complex and polygenic trait influenced by the photosynthetic source-sink relationship in wheat. The top three leaves, especially the flag leaf, are considered the major sources of photo-assimilates accumulated in the grain. Determination of significant genomic regions and candidate genes affecting flag leaf size can be used in breeding for grain yield improvement.

**Results:**

With the final purpose of understanding key genomic regions for flag leaf size, a meta-analysis of 521 initial quantitative trait loci (QTLs) from 31 independent QTL mapping studies over the past decades was performed, where 333 loci eventually were refined into 64 meta-QTLs (MQTLs). The average confidence interval (CI) of these MQTLs was 5.28 times less than that of the initial QTLs. Thirty-three MQTLs overlapped the marker trait associations (MTAs) previously reported in genome-wide association studies (GWAS) for flag leaf traits in wheat. A total of 2262 candidate genes for flag leaf size, which were involved in the peroxisome, basal transcription factor, and tyrosine metabolism pathways were identified in MQTL regions by the in silico transcriptome assessment. Of these, the expression analysis of the available genes revealed that 134 genes with > 2 transcripts per million (TPM) were highly and specifically expressed in the leaf. These candidate genes could be critical to affect flag leaf size in wheat.

**Conclusions:**

The findings will make further insight into the genetic determinants of flag leaf size and provide some reliable MQTLs and putative candidate genes for the genetic improvement of flag leaf size in wheat.

**Supplementary Information:**

The online version contains supplementary material available at 10.1186/s12864-023-09119-5.

## Background

Wheat (*Triticum aestivum* L.) is one of the most important cereal crops worldwide and provides about one-fifth of the calories consumed in the global diet [1]. There is a major concern that the risk of a global food crisis is increasing, due to the ever-increasing population, extreme climate changes, and reduction in arable land [2]. It is also estimated that an additional 1 billion tons of grain per year will need to be grown by 2050 to meet food demands [3]. To address this issue, wheat breeders are emphasizing trait-based breeding using genotype complementation with elite agronomic traits to accelerate grain yield improvement [4, 5]. Further improvement in wheat genetic gain can likely be achieved by the breeding for key yield-related agronomic and physiological traits [6].

Grain yield is a complex and polygenic trait that is influenced by the photosynthetic source-sink relationship that determines changes in carbohydrate synthesis, accumulation, and distribution, especially in mature leaves [7–11]. Recent studies have shown that delaying leaf senescence in plants can contribute to maintain source-sink relationships, resulting in higher grain yields [12–14]. In wheat, the top three leaves, especially the flag leaf, are considered the major sources of photo-assimilates that accumulate in the grain [11, 15]. The flag leaf contributes about 45 to 58% of total photosynthetic activity [16] and over 40% of assimilates during grain filling [17]. The orientation and size of flag leaves are important in plant breeding, because they affect plant canopy morphology and photosynthetic efficiency [18]. The size of the flag leaf, consisting of leaf length, width, and area, is an extremely important factor that determines leaf structure and yield potential [17, 19]. Therefore, it is of high priority to understand the genetic mechanisms underlying flag leaf traits in wheat.

Flag leaf size is a typical quantitative trait, controlled by polygenes and highly influenced by environmental factors [20–22]. Several efforts have been made to explain the genetic mechanisms underlying flag leaf size by two strategies of quantitative trait loci (QTL) mapping [23–25] and genome-wide association studies (GWAS) [10, 26–28] in wheat. However, the significance of these QTL mapping results is strongly influenced by the experimental conditions, the type and size of mapping populations, density of genetic markers, and statistical methods used [29, 30]. In this context, these identified QTLs are often not robust enough to be used directedly in wheat breeding for marker-assisted selection (MAS) [31]. Therefore, the discovery of major and robust QTLs and closely associated markers with high potential for molecular breeding remains a challenge [32].

As another method for integrating QTL information, the meta-QTL (MQTL) analysis provides an effective strategy for identifying major genomic regions governing traits, regardless of the genetic backgrounds and environments [33]. This method has been used to identify consensus regions by examining QTL data from independent studies for their effect and consistency across different genetic backgrounds and environments, and to refine and confirm QTL positions on a consensus map by using mathematical models [34]. In wheat, MQTL analysis has also been successfully used to identify consensus QTL regions for yield-related traits [35–38], drought and heat tolerance [39–42], disease resistance [43–46], grain quality traits [31, 47–49], root-related traits [50–52], and so on. Likewise, MQTL has also been widely used for the different quantitative traits in different species such as rice (*Oryza sativa* L.) [53–55], maize (*Zea mays* L.) [56–58], barley (*Hordeum vulgare* L.) [59], and cotton (*Gossypium hirsutum* L.) [60]. MQTL analysis examined relevant QTL studies and refined the confidence intervals (CIs) of QTLs or QTL clusters to identify more reliable QTLs [38]. With the release of the high-quality genome sequence of Chinese Spring [61], there is an unprecedented likelihood of using these public resources to uncover the molecular mechanisms that influence important wheat agronomic traits at the genetic level [62]. In the same way, numerous transcriptomic data of wheat are available on a user-friendly platform [63, 64]. For instance, MQTL analysis combined with transcriptome assessment for important quality traits in wheat was performed [47], which led to the identification of 110 MQTLs, and finally 44 candidate genes with high probability of association with quality traits. Saini et al.(2022) [65] identified 141 MQTLs out of 2852 major MQTLs for yield and related traits and further predicted 1202 candidate genes within major MQTL regions and 50 homologs of associated genes for yield from other cereals. Eighty-six MQTLs for yield-related traits were also identified from 381 original QTLs under different environmental conditions, of which 18 genes or gene clusters associated with these MQTLs were validated in this study [36].

The aim of the present study was to conduct a meta-analysis for flag leaf size from independent QTL mapping studies published in the last decades and to deepen the genetic architecture underlying flag leaf size by discovering putative genes and incorporating transcriptomic studies. The results will provide further insight into the genetic determinants for flag leaf size, and some reliable QTLs and putative candidate genes will be suggested to be employed for the genetic improvement of these traits in wheat.

## Results

### Quantitative trait loci controlling flag leaf size in wheat

**Fig. 1:**
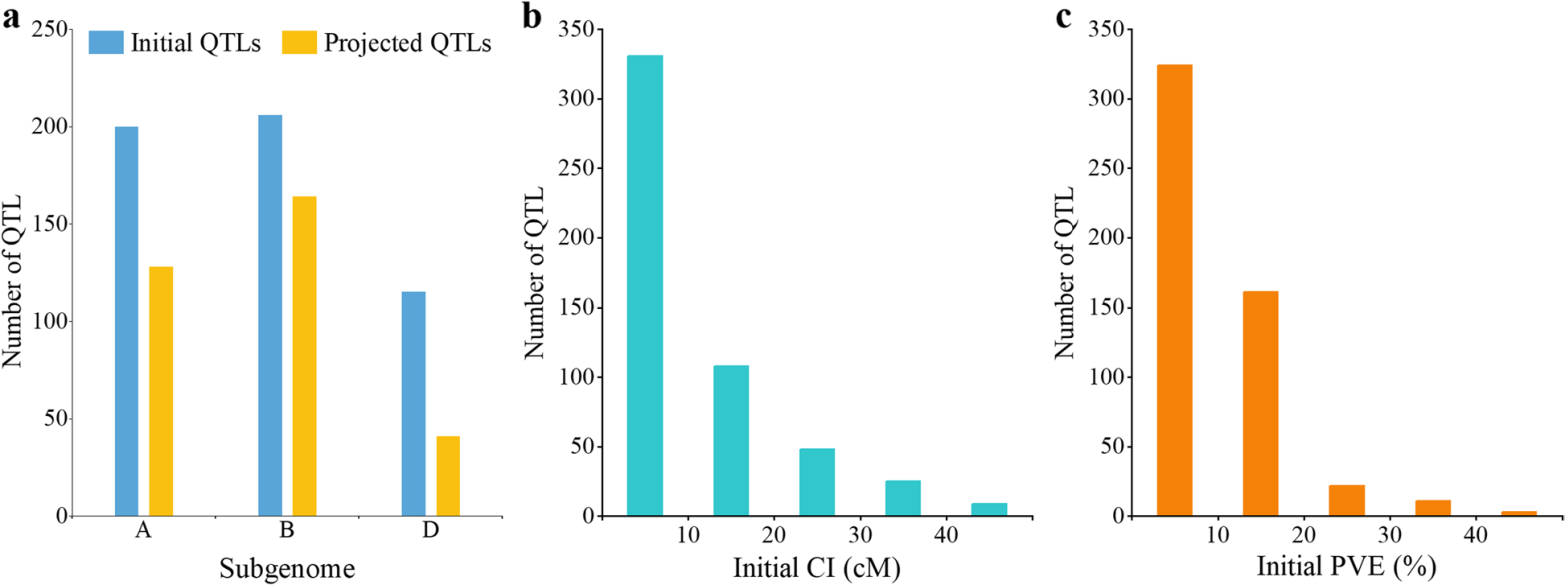
**a** Number of initial and projected QTLs. **b** Confidence intervals of the initial QTLs. **c** The individual PVEs of QTLs

A total of 31 studies published between 2008 and 2020, involving 34 recombinant inbred line (RIL) populations, three double haploid (DH) populations, and two backcross (BC) populations, were thoroughly reviewed to compile information on available QTLs (Table 1). As a result, 521 initial QTLs associated with flag leaf size were collected and distributed among all 21 wheat chromosomes. Of the earlier reported 521 initial QTLs, 38.39% (200) were distributed to subgenome A, 39.54% (206) to subgenome B, and only 22.07% (115) to subgenome D (Fig. 1a). Only 333 QTLs were successfully projected onto the consensus map (Fig. 1a and additional file 3), whereas the associated markers of the remaining 188 QTLs were absent from the consensus map or had a low phenotypic variation explained (PVE) value or large CI. The projected 333 QTLs were identified on all 21 chromosomes except 2D, 3A, and 5D. The highest number of projected QTLs was on subgenome B with 164 QTLs, whereas the lowest number of projected QTLs was on subgenome D with 41 QTLs (Fig. 1a). Correspondingly, the 95% CI varied from 0.04 cM to 55.14 cM, with approximately 63.53% (331) of the collected initial QTLs having a CI of less than 10 cM and 84.26% (439) having a CI of less than 20 cM (Fig. 1b). The PVE values for individual QTLs ranged from 1.05 to 54.38% with a mean of 10.23%. Only 39.92% (208) of the initial QTLs had PVE values greater than 10% (Fig. 1c).

**Table 1:** Details of previous QTL studies used for Meta-QTL analysis

No	Parents of population	Population size	Population type	Type and number of markers	References
1	Hua Pei 3 / Yumai 57	168	DH	SSR, EST-SSR (305)	[66]
2	Halberd / Cutter	64	RIL	SSR (170)	[67]
3	(Halberd / Karl 92) / Cutter	121	RIL	SSR markers and morphological marker (190)	[68]
4	Longjian19 / Q9086	120	RIL	SSR (405)	[69]
5	Weimai 8 / Luohan 2	302	RIL	SSR (348)	[70]
6	Nanda 2419 / Wangshuibai	230	RIL	EST-SSR (405)	[24]
7	Zardak / 249	130	RIL	SSR, EST-SSR, RAPD (71)	[71]
8	Xiaoyan 81 / Xinnong 1376	236	RIL	SSR (172)	[72]
9	Kenong9204 / Jing411	188	RIL	SSR (591)	[73]
10	Hanxuan 10 / Lumai 14	150	DH	SSR (395)	[74]
11	Ningchun 4 / Ningchun 27	128	RIL	SSR (291)	[75]
12	Yanda1817 / Beinong6	269	RIL	SSR, ET-SSR, SNP (2559)	[76]
13	Forno / Oberkulmer	226	RIL	SSR (182)	[77]
14	Zhou 8425B / Xiaoyan 81	102	RIL	SNP, SSR (6949)	[78]
15	Ningchun 4 / Drasdal	148	RIL	SSR (1000)	[79]
16	Lankao / Xiaoyan81	133	RIL	SSR (202)	[80]
17	Doumai / Shi 4185	275	RIL	SNP (11012)	[25]
	Gaocheng 8901 / Zhoumai 16	176	RIL	SNP (11979)	[25]
	Linmai 2 / Zhong 892	273	RIL	SNP (10443)	[25]
18	ND3331 / Zang1817	213	RIL	SSR (335)	[18]
19	CO940610 / Platte	185	DH	SSR, DArT, STS and protein based markers (462)	[81]
20	Weimai 8 / Luohan 2	179	RIL	DArT (576)	
	Weimai 8 / Yannong 19	175	RIL	DArT (576)	[82]
	Weimai 8 / Jimai 20	172	RIL	DArT (576)	
21	SeriM82 / Babax	167	RIL	SSR, AFLP and DArT (475)	[23]
22	Kenong9204 / Jing411	188	RIL	SNP (119566)	[83]
23	20,828 / Chuannong 16	199	RIL	SNP (119566)	[84]
24	WL711 / C306	206	RIL	SSR and STS (173)	[85]
25	Yanzhan 1 / Cayazheda 29	82	RIL	SNP (2059)	[86]
	Yanzhan 1 / Yunnanxiaomai	98	RIL		
	Yanzhan 1 / Yutiandaomai	93	RIL		
	Yanzhan 1 / Hussar	97	RIL		
26	(Shanghai 3 / Catbird) / Naxos	137	RIL	SSR (373)	[87]
27	20,828 / SY95-71	128	RIL	SNP, PCR-based markers (2529)	[88]
28	Lumai 14 / Jing 411	160	BC3F6	SSR (156)	[89]
	Lumai 14 / Shaanhan 8675	160	BC3F6	SSR (185)	[89]
29	Proteo / Chajia	97	RIL	SSR, SNP (2810)	[28]
30	Xiaoyan 8 / Xinong 1376	120	RIL	SNP (5531)	[90]
31	JingDong 8 / Aikang 58	207	RIL	SSR (149)	[91]

*SSR* single sequence repeat, *EST-SSR* experssed sequence tags-single sequence repeat, *RAPD* random amplified polymorphic DNA, *SNP* single nucleotide polymorphism, *DArT* diversity arrays technology, *AFLP* amplified fragment length polymorphism

### Projection of initial QTLs and identification of meta-QTL for flag leaf size

**Fig. 2:**
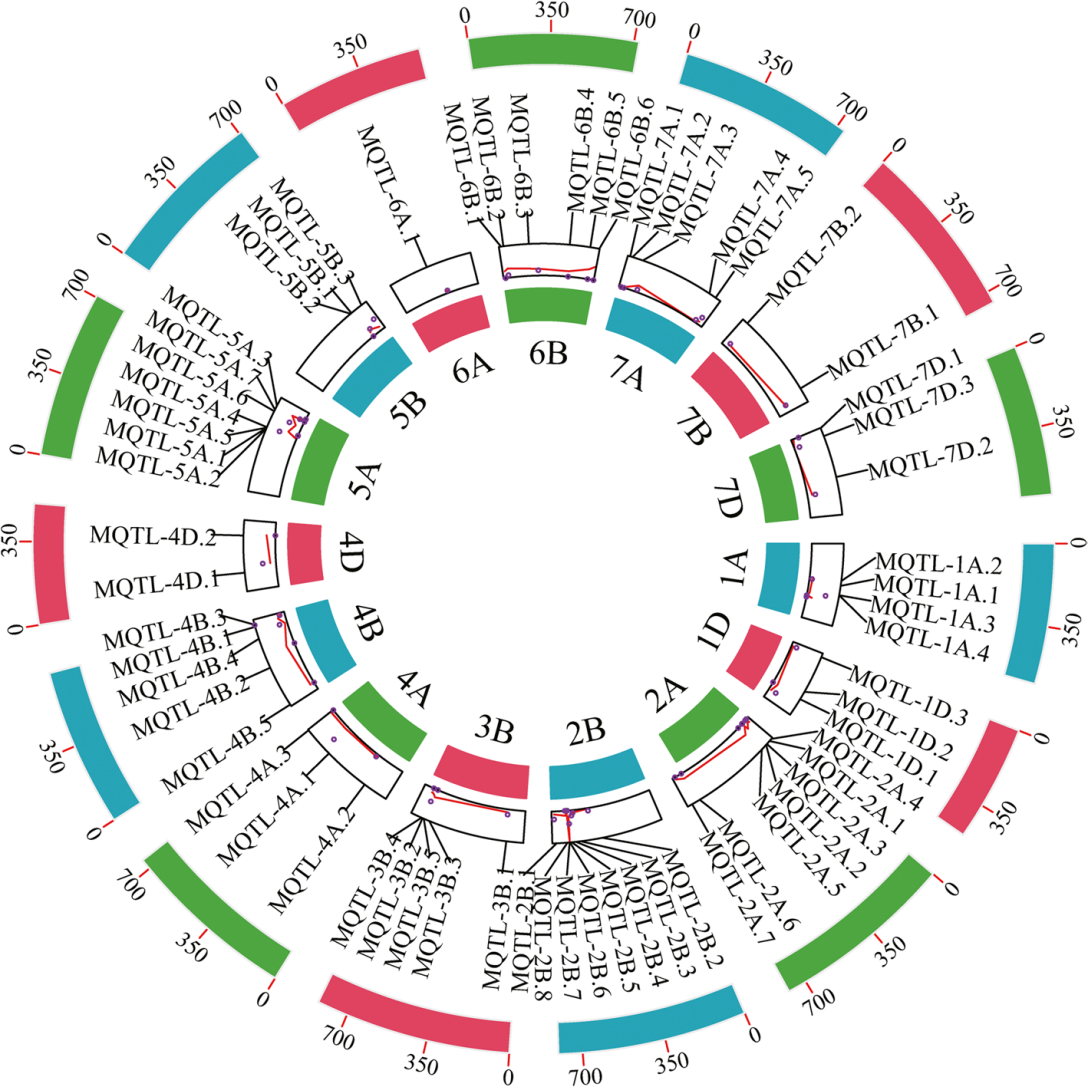
The chromosome distribution of 64 MQTLs for leaf size by MQTL analysis. The circles from outside to inside represent the physical chromosome distance (Mb), the position of 64 MQTLs, and the number of initial QTLs, respectively

**Fig. 3:**
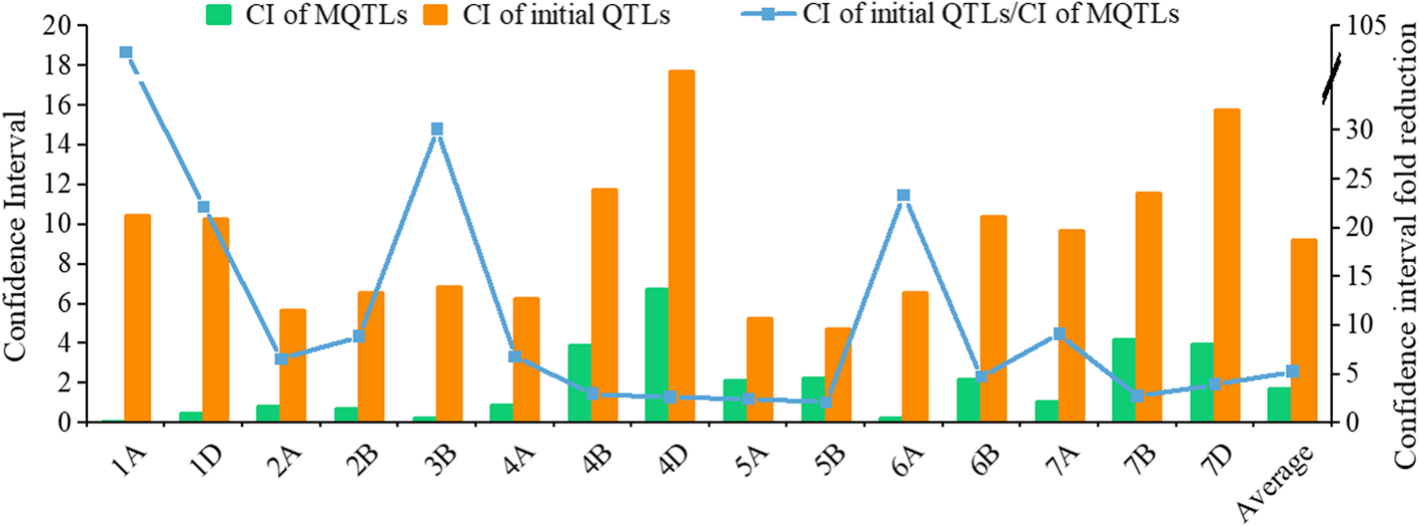
Comparison of mean CI for initial QTLs and MQTLs

A total of 64 MQTLs were generated from the 333 projected QTLs based on the criteria of the lowest model value and at least two overlapping initial QTLs (Fig. 2, Table S1). The 95% CI of the identified MQTLs, ranging from 0.02 to 18.05 cM with an average of 1.74 cM, was 5.28-fold reduction than that of the initial QTLs (Fig. 3). This indicated that these MQTLs were mapped more accurately. In addition, there were significant differences in the average CIs of the MQTLs between different chromosomes. For example, the average CI of MQTLs on chromosomes 1A, 3B, 6A and 1D decreased 102.28-, 30.16-, 23.38- and 22.14-fold, respectively (Fig. 3). Based on the comparison of the flanking marker sequences, the physical locations of all 64 MQTLs ranged from 1.64 Mb (MQTL 2B.6) to 206.35 Mb (MQTL 5A.2) (Table S1). These MQTLs, were then selected for further identifying putative candidate genes. Of the MQTLs identified, 13 were core MQTLs, such as MQTL2A.1, MQTL 2A.2, MQTL 2A.3, MQTL 2A.5, MQTL 2B.4, MQTL 2B.6, MQTL 2B.8, MQTL 3B.4, MQTL 3B.5, MQTL 5A.4, MQTL 5B.3, MQTL 6B.6, and MQTL 7A.5, met the previously established criteria for the search for candidate genes in the databases available. Moreover, the physical distances of the core MQTLs ranged from 1.64 to 18.77 Mb, while the genetic distance ranged from 0.02 to 0.9 cM (Table 2).

**Table 2:** Depiction of 13 core MQTLs identified for flag leaf size in wheat

MQTL	Initial QTLs	Traits	CI (cM)	Genetic interval (cM)	Physical interval (bp)	Physical distance (Mb)
MQTL-2A.1	2	FLW(1),FLA(1)	0.06	34.37-34.43	31,025,604-40,589,528	9.56
MQTL-2A.2	2	FLL(1),FLA (1)	0.21	36.54-36.75	67,712,263-78,329,436	10.62
MQTL-2A.3	2	FLL(1),FLA(1)	0.35	37.58-37.93	59,553,716-78,326,762	18.77
MQTL-2A.5	2	FLL(1),FLW(1)	0.41	42.81-43.22	112,742,680-122,146,435	9.4
MQTL-2B.4	3	FLL(1),FLW(1),FLA(1)	0.12	80.90-81.02	632,921,191-636,481,655	3.56
MQTL-2B.6	2	FLL(1),FLA(1)	0.25	95.58-95.83	676,172,474-677,808,160	1.64
MQTL-2B.8	2	FLL(1),FLW(1)	0.3	103.29-103.59	693,069,555-694,997,337	1.93
MQTL-3B.4	2	FLL(1),FLW(1)	0.1	89.04-89.14	750,141,842-760,139,108	10
MQTL-3B.5	7	FLL(4),FLW(2),FLA(1)	0.42	82.82-83.24	732,824,415-738,985,172	6.16
MQTL-5A.4	9	FLL(3),FLW(2),FLA(4)	0.9	78.13-79.03	644,132,169-663,836,794	19.7
MQTL-5B.3	7	FLL(3),FLW(2),FLA(2)	0.02	145.21-145.23	670,524,595-690,225,662	19.7
MQTL-6B.6	2	FLL(2)	0.11	153.92-154.03	717,862,165-719,732,713	1.87
MQTL-7A.5	5	FLL(2),FLW(1),FLA(2)	0.42	64.33-64.75	694,930,915-701,309,255	6.38

*MQTL* Meta-QTL, *FLL* flag leaf length, *FLW* flag leaf width, *FLA* flag leaf area, *CI* the confidence interval

### MQTLs matching MTAs from previous genome-wide association studies

**Fig. 4:**
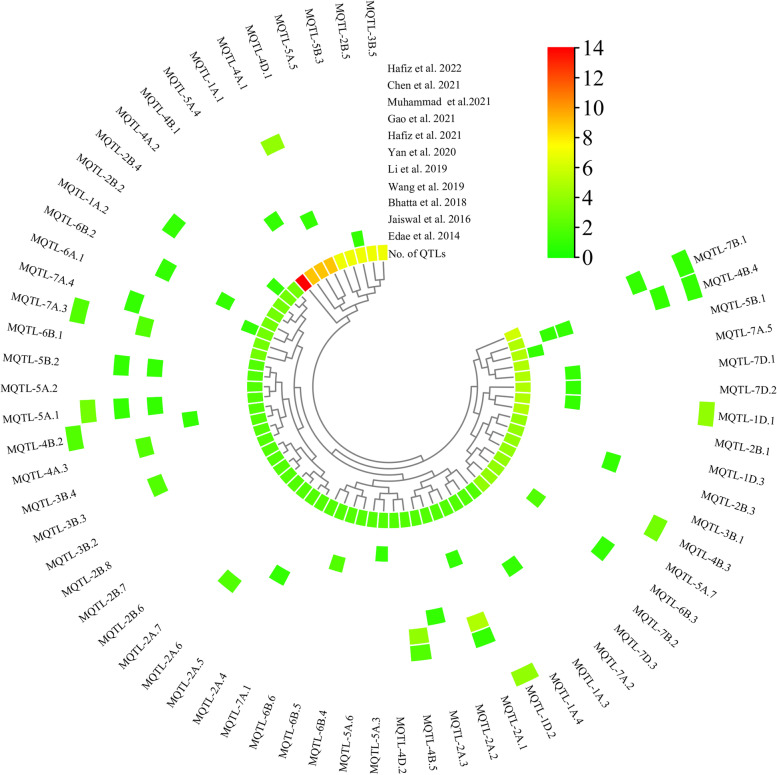
Validation of MQTL by MTAs in wheat traits associated with flag leaves from GWAS with 11 different natural populations

The physical positions of the MQTLs identified in this study and the marker trait associations (MTAs) from 11 previous studies were used for comparison to further determine the accuracy of MQTL for flag leaf size (Fig. 4, Table S2). Accordingly, 51.56% (33/64) of the identified MQTLs were co-located with 77 SNP peak positions early reported in GWAS for leaf size in wheat. This indicated that only half of the MQTL regions could be validated by the MTAs. The number of MTAs colocalized for each MQTL also varied from one to seven in 11 GWAS studies. Each of these 33 MQTLs was colocalized with at least one MTA. Of these, MQTL-2A.1, MQTL-4B.5 and MQTL 4A.1 were colocalized with 7, 6 and 5 MTAs, respectively.

### Putative genes and in silico gene expression analysis

In this study, three approaches were developed to identify putative candidate genes associated with leaf size in wheat. An exhaustive search for known rice genes associated with flag leaf traits resulted in a collection of 97 candidate genes (Table S3) that were used to identify their corresponding homologs in wheat. Only 41 genes of these were identified within 22 MQTL regions (Table S4). These genes were reported to affect leaf-related traits of rice through a variety of proteins/products, such as auxin response factor, zinc finger protein, probable transcription factor, cyclin-dependent kinase inhibitor, growth regulating factor, and so on. The number of putative genes within each MQTL varied from one to five, with an average of 1.86 genes per MQTL. We identified 2262 genes within MQTL regions, including 41 genes with corresponding homologs for the leaf traits in rice (Table S4), and 2221 putative genes after eliminating duplicate genes in overlapping MQTLs (Table S5). Most putative genes (278 genes) were identified within the confidence region of MQTL-5B.3, whereas only one gene was found on chromosome 6A. These genes with similar function included 183 putative genes for F-box-like domain proteins, 78 for protein kinases, 48 for BTB/POZ domain-containing proteins, 33 for leucine-rich repeat domain proteins, 25 for glycosyltransferase family proteins, and 21 for cytochrome P450 proteins, etc. (Fig. S1).

**Fig. 5:**
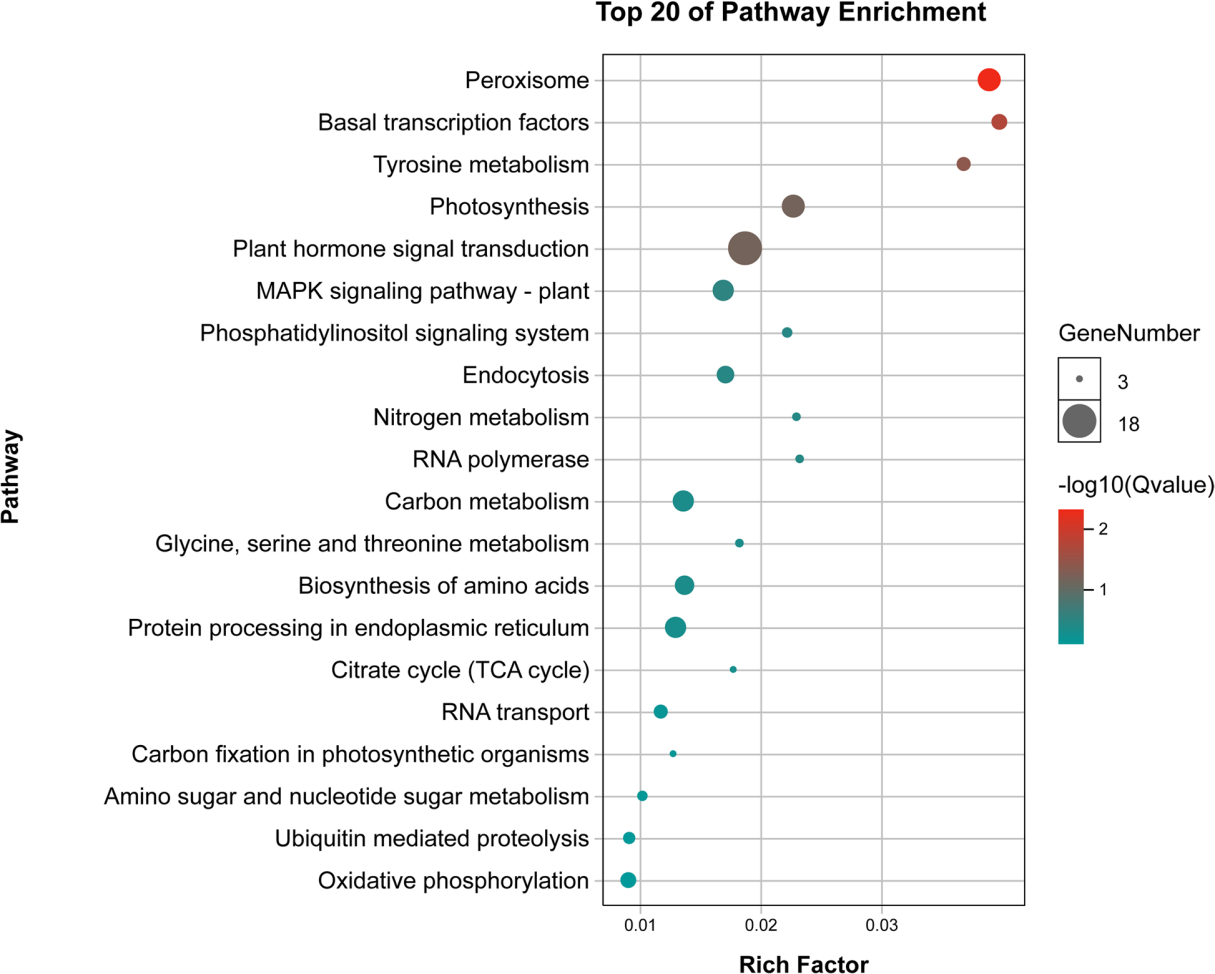
Top 20 KEGG enrichment pathways for 134 putative candidate genes from MQTL regions

**Fig. 6:**
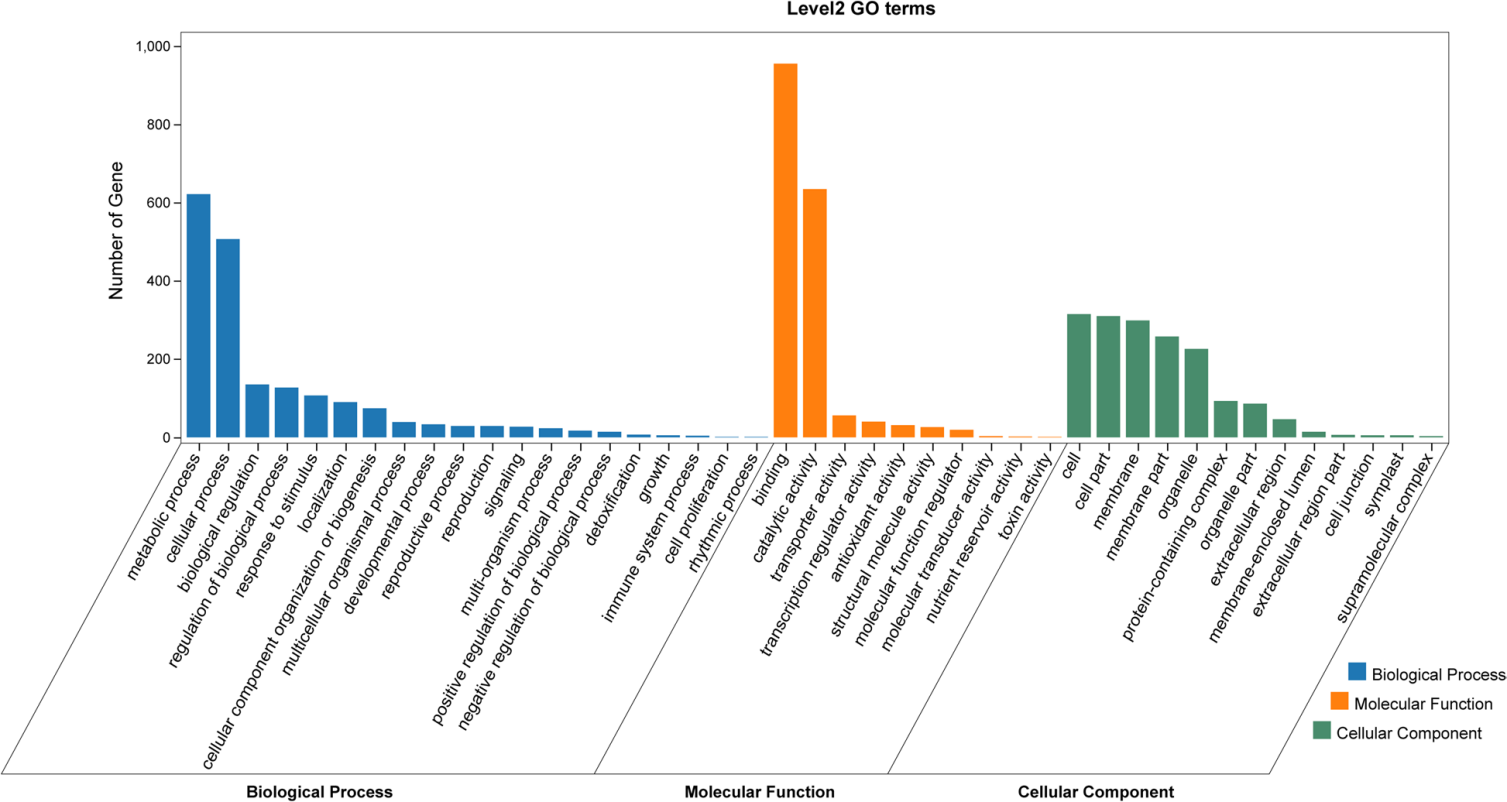
Level 2 GO terms for 134 putative candidate genes from MQTL regions

Gene ontology (GO) enrichment and Kyoto Encyclopedia of Genes and Genomes (KEGG) pathway analyses were performed to determine the functional classification of the identified genes. KEGG enrichment analysis revealed that these putative genes were highly involved in the peroxisome, basal transcription factor, and tyrosine metabolism pathways, with the greatest number of putative genes in the peroxisome pathway (Fig. 5). GO analysis revealed a range of GO terms, of which some of the most important and abundant GO terms included those involved in all three categories. The most enriched GO terms related to biological processes involved metabolic processes (621 genes) and cellular processes (506 genes). The most enriched GO terms related to molecular functions involved binding (955 genes) and catalytic activities (634 genes). Regarding cellular components, genes were mainly enriched in the cell (314 genes) and in cell parts (309 genes) (Fig. 6).

Some critical genes controlling leaf size that emerged from enrichment analysis of GO and KEGG pathways were considered as selected genes for further in silico expression analysis.

**Fig. 7:**
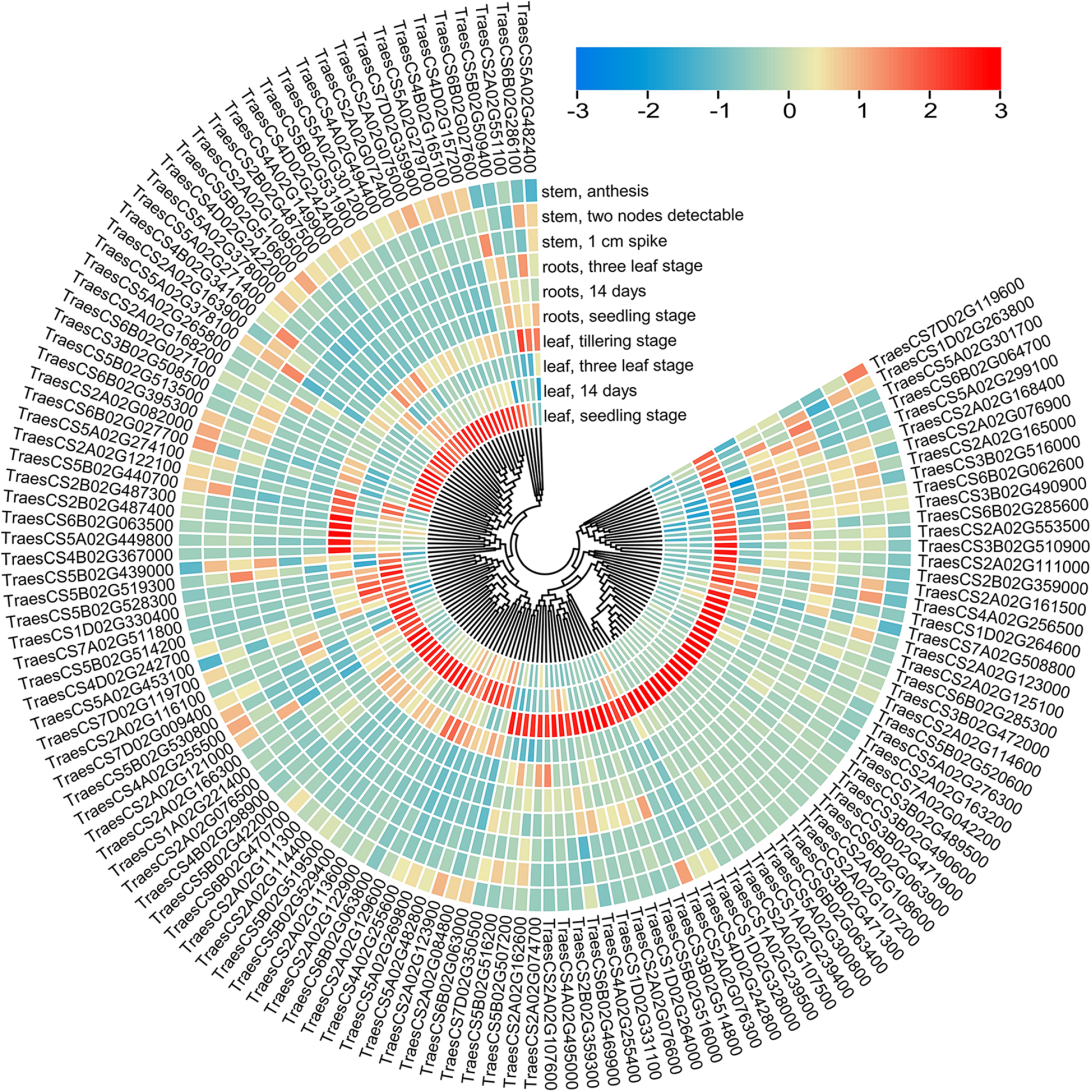
Expression characteristics of 134 putative candidate genes in five different tissues. Transcriptome data were downloaded from expVIP

The expression analysis of the available genes revealed that 134 genes with > 2 transcripts per million (TPM) were highly and specifically expressed in the leaf (Fig. 7, Table S6). Based on their expression, these genes could be divided into three classes (Fig. 7). In class I, the genes showed high expression in leaf during seedling and tillering stage, while in class II, the genes showed high expression in leaf at 14-day development stage and finally in class III, the genes showed high expression in leaf at three-leaf stage. Despite in the same tissues, the expression of the genes (e.g.*, TraesCS2A02G072400, TraesCS4B02G36700, TraesCS6B02G063400* and so on) varied in different growth stages (Fig. 7). Consequently, the expression analysis of 134 putative candidate genes at different developmental stages allowed us to realize their potential roles in seedling leaf size, which were hypothesized to influence leaf size at the adult plant stage.

## Discussion

### Identification of key MQTL regions through meta-analysis

Extensive studies on QTL mapping of yield and other important agronomical traits in wheat have been conducted in recent decades. Nevertheless, most QTLs identified in these studies are each associated with a long CI and low PVE. This made these QTLs less useful for the marker-assisted breeding. In contrast, MQTLs with a narrow CI and a relatively high PVE are more compelling in proving useful for molecular breeding [65]. It was also found that the results of the MQTL analysis were significantly and positively correlated with the quality of the QTL mapping results [92]. In addition, new QTLs are regularly added to the databases as molecular genetics and QTL mapping methods continue to evolve. Therefore, it is very important to keep up with this pace to integrate new QTLs into more stable and reliable MQTLs [47]. In the present study, 521 initial QTLs were collected from 31 studies between 2008 and 2020 to identify genomic regions associated with flag leaf size in wheat (Table 1). Compared with subgenomes A and B, the subgenome D had a lower number of QTLs, which was consistent with previous MQTL analyses for grain yield and other yield-related traits [32, 36, 65, 92]. Only about 24.4% of the QTLs were mapped on subgenome D, while about 75.6% were found on subgenomes A and B (Fig. 1a). One possible reason for this phenomenon could be that the subgenome D has a low level of DNA polymorphism. Compared to the diploid progenitor species *Aeegilops tauschii*, very low genetic diversity has been observed for the subgenome D of wheat [93]. Meanwhile, there has also been a limited gene flow from *Aegilops tauschii* to cultivated wheat [94].

For the 64 MQTLs identified in the present study, the CI of the identified MQTLs with an average of 1.74 cM reduced 5.28-fold compared with the mean value of the corresponding initial QTLs (Fig. 3). In a similar study, the discovery of 13 MQTLs with an average CI of 13.6 Mb for the initial QTLs and 6.01 Mb for the MQTLs was found to be 2.26-fold reduction than that of the initial QTLs for drought tolerance in bread wheat [32]. Moreover, the definitive physical position of the 64 MQTLs in the present study was obtained by the publication of the wheat genome reference sequence of Chinese Spring, where the physical position of the identified 64 MQTLs varied from 1.64 Mb (MQTL 2B.6) to 206.35 Mb (MQTL 5A.2). Interestingly, 48 of the identified MQTLs contained a 95% genetic CI below 2 cM (Table S1).

It is widely accepted that optimizing flag leaf morphology, including the leaf length, width and area are important determinants to increase yield in wheat [82, 84, 95, 96]. Generally, leaf size is controlled by two main dimensions: leaf length and width which are sensitive to environmental factors [97, 98]. Previous studies have detected significant and positive correlation between FLW and FLA, also found that FLW was more crucial than FLL in determining FLA [73, 84]. Of the 64 MQTLs identified in the current study, 45 MQTLs were detected associated with FLA, of which 32 MQTLs correlated with FLW and 35 MQTLs related to FLL. It seemed that there was much the same of their contribution on FLA. Moreover, there were 22 MQTLs all associated with FLL, FLW and FLA. The comparison of initial QTLs PVE of 22 MQTLs found that 14 MQTLs possessed higher PVE with FLW than FLL. Similar to early studies, this result further demonstrated that FLW as the major contributor had more influences on FLA. Also, Li et al. (2018) reported that FLW can be used to select lines with large KN which is one of the main components of grain yield [25]. Therefore, individuals with wider flag leaves should be selected to increase FLA and also increase yield potential in wheat breeding programs.

It has been demonstrated that several QTL intervals for flag leaf traits were mapped to the same or similar chromosomal regions for yield-related traits in the previous studies. For example, Ma et al. (2020) found that the interval of *QFll.sicau-2D.3, QFlw.sicau-2D and QFla.sicau-2D* were closely related to QTL for spikelet number per spike, plant height (PH), anthesis date, thousand-grain weight (TGW), spike length (SL) and kernel number per spike (KN) [84]. Liu et al. (2018) confirmed that *QFLA-4B.1* and *QFLA-4B.2*, were detected close to marker *Xbarc20*, which was also found to co-localize with QTL for PH, SL, spike number per plant, KN, GW, and TGW [18]. In addition, 34 MQTLs identified in the current study had their physical positions almost coincidence with those physical positions of MQTLs reported in three recent studies for yield-related traits [38] (Table S1). Given that MQTL for flag leaf traits that showed consistent relationships with yield-related traits, these MQTLs with a higher level of confidence may be described as pleiotropic regions which can effectively improve breeding efficiency for multiple traits.

There were 13 core MQTLs were selected basing on the preferred criteria of at least two overlapping initial QTLs with a physical distance < 20.0 Mb and a genetic distance < 1.0 cM [44] (Table 2), a higher level of confidence for further analysis and for identification of candidate genes. These 13 core MQTLs showed a smaller genetic CI (0.28 cM) compared with the initial QTLs (9.18 cM) with 32.79-fold reduction. Of these, MQTL-2A.1, MQTL-2A.3, MQTL-2B.4, MQTL-5A.4 and MQTL-5B.3 were validated by the MTAs. As for the 134 genes obtained via transcriptome and functional annotation, 39 genes were identified within the regions of 13 core MQTLs. It was worth mentioning that nearly half of (18/39) genes available from five core MQTL regions verified by the MTAs. Some of the significant features of these 13 core MQTLs detected in this study were described as follows: (i) They showed stability under different environments: MQTL-1A.1 consisted of nine initial QTLs for flag leaf length, width, and area with an average PVE of 6.54% from six different populations [22, 28], suggesting that MQTL-1A.1 exhibits strong stability for the flag leaf size trait. Apart from the above core MQTLs, the other core MQTLs also showed high stability under different environments. (ii) There were multiple core MQTLs accounting for the same traits. Except for MQTL-2A.1, 12 of the 13 core MQTLs were all based on the initial QTLs for flag leaf length. Likewise, MQTL-2A.1, MQTL-2A.2, MQTL-2A.3, MQTL-2B.4, MQTL-2B.6, MQTL-3B.5, MQTL-5A.4, MQTL-5B.3, and MQTL-7A.5 were composed of the initial QTLs for the flag leaf area trait (Table 2). These core MQTLs, not based on only one trait, were apparently more robust than initial QTLs. (iii) The core MQTLs showed pleiotropic effect. All 13 core MQTLs were responsible for controlling more than two traits. Of these, MQTL-5A.4 derived from nine initial QTLs, followed by MQTL-5B.3 and MQTL-3B.5 derived from seven initial QTLs for control of multiple traits (Table 2), suggesting that these MQTLs may represent a complex genomic region for control of more than one trait.

### Potential candidate genes associated with leaf size in meta-QTL regions

To support the location of the MQTLs identified in this study, an extensive literature search was conducted to identify known genes within MQTL regions. For example, Siddiqui et al. (2021) identified two candidate genes *TraesCS4B02G293600* and *TraesCS4B02G293700* on wheat chromosome 4 B[99]. They were strongly expressed in leaves and stems as well as under drought stress conditions, suggesting that the two genes are involved in photosynthetic pathways and drought tolerance mechanisms. The two genes were also located in the MQTL-4B.4 region identified in the present study. Liu et al. (2018) found that the gene *Ppd-D1* regulated leaf growth by controlling photoperiod, and its physical location was also found in the MQTL-5B.2 region in the current study [100]. When comparing the MQTLs identified in this study with the wheat gene *TaCHLI-7A*, encoding the protein CHLI involved in the biosynthesis of chlorophyll in common wheat [101], the gene was also located in the MQTL-7A.4 region. In addition, Muhammad et al. (2021) predicted 18 candidate genes for flag leaf length in wheat [27], where six of the predicted genes *TraesCS5A01G487600*, T*raesCS5A01G487700*, T*raesCS5A01G487800*, *TraesCS5A01G487900*, *TraesCS5A01G488000*, *TraesCS5A01G488100*, T*raesCS5A01G488200* were located with the MQTL-5A.5 region in this study.

Another important finding in the present study was that 2262 putative genes related to flag leaf size were identified within the MQTL regions and showed the spatiotemporal and specific expression pattern (Table S4, Table S5). These candidate genes mainly encode the F-box-like domain proteins, protein kinases and BTB/POZ domain-containing proteins. The F-box protein FBX92 played a role in regulating leaf growth in *Arabidopsis*, where FBX92 regulates the rate of cell division [102]. PINOID kinase may actually function by influencing auxin accumulation and distribution leading to influence auxin metabolism and signaling indirectly, and finally suggest a role in leaf development as well in *Arabidopsis* [103]. The candidate genes associated with wheat leaf size within the MQTL regions were identified through the analysis of transcriptome data. Given the close evolutionary relationship between *Gramineae* species genomes [104], candidate genes with unknown functions in the wheat genome were evaluated in the MQTL regions of our study based on their orthologous genes in the rice genome [48]. For example, eight wheat homologs for rice genes, *TraesCS1D02G393900, TraesCS1D02G394000, TraesCS1D02G394100, TraesCS4B02G064000, TraesCS5B02G508800, TraesCS7A02G059000, TraesCS7B02G484200,* and *TraesCS7D02G409700* were predicted in the early study [92], which were overlapped with six MQTLs identified in the present study. Of these, the first three genes were all present on MQTL-1D. 1, which was formed from five initial QTLs from three different populations. The last five genes were located on the MQTL-4B.5, MQTL-5B.3, MQTL-7A.1, MQTL-7B.1, and MQTL-7D.2, respectively. The involvement of these eight homologous genes in the different biological processes was associated with leaf size and chlorophyll content in rice, suggesting that these genes may be involved in the regulation of leaf size in wheat.

In addition, we annotated 2262 genes using GO or KEGG analysis (Figs. 5 and 6). KEGG and GO pathway enrichment analysis revealed that these putative genes were highly involved in the peroxisome, basal transcription factor, tyrosine metabolism, photosynthesis and plant hormone signal transduction pathways. Peroxisome get involved in the photorespiration and the synthesis of phytohormones, which are important for signaling pathways, including jasmonic acid, auxin, and salicylic acid [105, 106]. The *CFL2* regulated by transcription factor *Roc5*, encoding a cytochrome P450 protein, is involved in the regulation of flag leaf shape by influencing epidermis and cell wall development [107]. Tocochromanols and plastoquinone produced in the tyrosine biosynthetic pathways are essential metabolites produced in all plants and other photosynthetic organisms [108]. Plastoquinone is required for photosynthesis as an electron carrier, an enzyme involved in carotenoid biosynthesis and is a cofactor of phytoene desaturase. Tocopherols have unexpected roles in photo-assimilate transport [109, 110]. Herein, a total of 134 putative genes with TPM > 2 in the robust and stable MQTL regions were listed based on significant gene expression in the leaf that may potentially affect leaf size in wheat (Table S6). For example, *TraesCS4A02G149900, TraesCS4B02G165100*, and *TraesCS4D02G157200*, encoding an ATP-dependent proteolytic subunit of Clp protease, were specifically expressed at the leaf seedling stage, while their homologous *NAL9* genes caused reduced cell number in the lateral direction due to a significant reduction in the total number of vascular bundles in rice [111]. *TraesCS4B02G341600*, encoding a cytochrome P450 family protein, was also specifically expressed at the leaf seedling stage. Its homologous gene *sd37* in rice was confirmed to encode the putative cytochrome P450 protein CYP96B4, and the sd37 transgenic leaves were smaller than those of the wild type, reflecting a decrease in cell number in the mutant [112]. *TraesCS7D02G350500*, encoding β-ketoacyl-CoA synthase, was strongly expressed at the third leaf stage. In rice, its homologous gene *WSL1* showed a pleiotropic phenotype, including reduced growth and shortened leaves [113]. Although the relationship between these genes and leaf size in wheat has not been reported, their homologous genes have been shown to be involved in the regulation of leaf size in rice. In addition, 17 genes with TPM > 2 enriched in the peroxisome, photosynthesis, basal transcription factors and plant hormone signal transduction pathway. This suggests that these 134 putative candidate genes may have potential effects on leaf size regulation in wheat.

## Conclusions

In this study, we deciphered key genomic regions controlling flag leaf size in wheat by integrating MQTL analysis and in silico transcriptome assessment. The 333 initial QTLs were successfully projected on the reference genetic map and refined into 64 MQTLs. Of these, 13 core MQTLs showed the mean CI was 32.79-fold reduction than initial QTLs and five core MQTLs were validated by the MTAs, suggesting as potential loci in MAS for flag leaf size in wheat. The 2262 putative candidate genes were mined within the MQTL regions by the genomic sequence comparison, where 134 candidate genes with more than 2 TPM were highly and specifically expressed in the leaf by in silico gene expression analysis. This suggested that, if these key MQTL regions and candidate genes will be further validated through biological experiment strategies, they have great application potential in molecular genetic improvement of flag leaf size in wheat.

## Methods

### Bibliographic collection of QTLs for flag leaf size and construction of reference map for QTL projection

For QTLs controlling for flag leaf size, a comprehensive bibliographic collection was performed using PubMed (http://www.ncbi.nlm.nih.gov/pubmed), Google Scholar (https://scholar.google.com/) and China National Knowledge Infrastructure (https://www.cnki.net/). QTL projection were conducted if all required information were available. For each study, QTL information collected included: (i) QTL name; (ii) three flag leaf related traits, including flag leaf length (FLL), flag leaf width (FLW) and flag leaf area (FLA); (iii) closely related flanking markers; (iv) position of the peak and associated 95% CI; (v) type and size of lines in the mapping population; (vi) LOD score; and (vii) the phenotypic explained variation (PVE) or *R*^*2*^ values of the QTLs. In cases where the log of odds ratio (LOD) and *R*^*2*^ values were missing for some QTLs detected in previous studies, they were assumed to be 3 and 10, respectively [44, 53]. When the peak position was missing, the midpoint between the two flanking markers was treated as the position [92]. In addition, for the initial QTLs that were missing flanking markers and CIs, the CIs were recalculated according to population type and size using the following standard formula: (i) F_2_ and backcross population, CI = 530/ (N× *R*2); (ii) recombinant inbred line (RIL) population, CI = 163/ (N× *R*2); and (iii) doubled haploid population, CI = 287/ (N× *R*2). Here, 530, 163, and 287 are the population-specific constants obtained from different simulations [114, 115], N is the size of the mapping population used for QTL analysis, and *R*^*2*^ is the phenotypic variation explained by QTL [32].The main markers used to generate genetic linkage maps in QTL mapping studies include Simple Sequence Repeat (SSR), Diversity Arrays Technology (DArT), and Single Nucleotide Polymorphism (SNP) markers [36]. The genetic reference map obtained from two dense genetic maps [51, 116] was integrated as a high-density reference map [35]. This map contained 14,548 markers, including SSR, DArT, SNP and other types of markers, with a total length of 4813.72 cM, ranging from 155.6 cM to 350.11 cM in the 21 linkage groups. The map was used as reference map for projection of individual QTLs identified in independent populations [48].

### QTL projection and meta-QTL analysis

The initial QTLs data, the associated individual genetic maps from previous independent studies, and the reference genetic map were used as input files to create a consensus map and further perform the MQTL analysis [92]. BioMercator v4.2 software was used for projection [117, 118], the initial QTLs and the information of each QTL, for instance, CI, peak position, LOD score and and *R*^*2*^ were projected onto a reference map [117]. QTLs were discarded when they could not be projected onto the consensus map and those mapped to positions outside the consensus map [32].

After projection, MQTL analysis was performed on each chromosome using BioMercator v4.2 software [117, 118] via the Veyrieras two-step algorithm [118, 119]. Two different approaches were used based on the number of initial QTLs on each chromosome. In the first approach, the meta-analysis proposed by Goffinet and Gerber (2000) [119] was applied when the number of initial QTLs on a chromosome was less than 10. Based on this approach, the best MQTL model with the lowest AIC values for QTL integration and identification of consensus MQTL positions in BioMercator v4.2 software was selected. On the other hand, if the number of QTLs in a chromosome was at least 10, the second method proposed by Veyrieras [120] was used. According to this approach, meta-analyses were performed for individual chromosomes using a two-stage approach available in the software. In the first step, the collected QTLs on individual chromosomes are clustered using default parameters. The number of potential MQTLs per chromosome is then estimated based on the following five selection criteria, including Akaike information criterion (AIC), corrected Akaike information criterion (AICc), Akaike information criterion 3 (AIC3), Bayesian information criterion (BIC), and approximate weight of evidence (AWE). A QTL model that had the lowest values of the selection criteria was considered the best optimal model for the next step of meta-analysis. In the second step, the 95% CI and the positions of each MQTL were determined according to the optimal model selected in the previous step. The QTLs were integrated so that the peak position of the initial QTLs was in the MQTL CI [34], whereas the MQTLs without the minimum AIC values were to be discarded.

### Identification of putative genes in MQTL regions

The putative genes are located within the regions identified basing on the positions of the flanking markers of the MQTL (or the marker closest to the flanking markers) [32]. After identifying the MQTLs, the next step was to find the flanking markers within the target MQTLs based on the 95% CI and the positions of each MQTL. The physical positions of each MQTL were obtained based on the positions of flanking markers which were searched in the Triticeae Multi-omics Center (http://wheatomics.sdau.edu.cn) annotated by IWGSC_v1.1_HC_gene. When the physical positions of the flanking markers were not found, the GrainGenes database (https://wheat.pw.usda.gov/GG3) or the DArT database (https://www.diversityarrays.com) were used to obtain their sequences. The sequences information was then aligned to the wheat reference genome in the Triticeae Multi-omics Center (http://wheatomics.sdau.edu.cn), using the BLASTN program to find the physical position of flanking markers [92].

MQTLs are considered potential genomic regions that are likely harbor putative genes for the traits [121]. Based on other meta-analyses published in recent years, three methods have been used to identify putative genes within MQTL regions [44, 92]. (i) In the first method, the strategy of orthologous comparison between wheat and rice was used to identify the major putative genes in the MQTL regions. For this purpose, the China Rice Data Center (https://www.ricedata.cn/gene/) was manually used to identify the genes for flag leaf associated traits in rice. In addition, the homologous genes of wheat were retrieved from the Triticease-Gene Tribe (http://wheat.cau.edu.cn/TGT/) based on the IWGSC RefSeq v1.1. (ii) To further refine the MQTL, those with at least two overlapping initial QTLs with a physical distance < 20.0 Mb and a genetic distance < 1.0 cM, referred to as core MQTLs, were selected from the second approach. (iii) The peak physical positions of the remaining MQTLs were calculated using 1-Mb region on each side of the MQTLs for mining relevant genes within the MQTL regions. The peak physical position of the MQTLs was calculated according to the method proposed by Saini [65]. Both the original and estimated range of physical positions were then entered into the search toolbox of the “Gene” in the WheatGmap database [122] to obtain details of gene models (locus ID information and functional descriptions) corresponding to MQTL regions.

### Verification of MQTLs by GWAS and known wheat genes within MQTLs

To further validate the accuracy of the discovered MQTL regions, available genome-wide association studies for flag leaf size were reviewed to search for MTAs that could be compared with the MQTLs identified in this study. Considering the relatively large linkage disequilibrium decay in wheat (approximately 5 Mb), the MTAs obtained from GWAS near MQTLs in the 5 Mb physical region were considered to be related to MQTLs [92]. The physical positions of known genes associated with flag leaf size within MQTLs were obtained from the Triticeae Multi-omics Center (http://wheatomics.sdau.edu.cn). Subsequently, the physical positions of these genes were compared with the genomic regions of the MQTLs to identify genes that might correspond to individual MQTLs [65].

### Expression of candidate genes within MQTL regions

Gene expression analysis examines how genes are transcribed to produce functional products such as RNA or proteins [47]. The GENEDENOVO cloud platform (https://www.omicshare.com) was used to perform the GO and KEGG analysis. For transcriptional expression analysis, the Expression Visualization and Integration Platform (expVIP, http://www.wheat-expression.com) with expression data from 18 tissues throughout the wheat growth period [64, 123] was used in this study. Only candidate genes showing at least 2 TPM expression were considered [124]. The expression characteristics of candidate genes were displayed by the heat map of TPM using the TBtools software [125]. In this study, the tissues and their corresponding stages were leaves at seedling, 14-day, three-leaf, and tillering stages; roots at seedling, 14-day, and three-leaf stages; stems at 1-cm spike, two-node, and anthesis stages.

## Supplementary Information

**Additional file 1: Table S1.** Description of 64 MQTLs identified for flag leaf size. **Table S2.** The GWAS analysis on flag leaf related traits used in this study. **Table S3.** The details of 97 rice known genes. **Table S4.** The 41 wheat homolog genes of 35 rice known genes located in MQTL. **Table S5.** The information of 2221 genes identified in 64 MQTL regions. **Table S6.** Summary of 134 putative candidate genes exhibiting significant expression (TPM > 2) within MQTLs.

**Additional file 2: Figure S1.** Frequency of candidate genes in each of 16 different proteins associated with flag leaf traits.

**Additional file 3.** The information of consensus map.

## Data Availability

The relevant data and additional information are available in the supplementary files.

## Abbreviations

AIC: Akaike information criterion
AICc: Corrected Akaike information criterion (AICc)
AIC3: Akaike information criterion 3
AWE: Approximate weight of evidence
BC: Backcross populations
BIC: Bayesian information criterion
CI: Confidence interval
DArT: Diversity arrays technology
DH: Double haploid populations
expVIP: Expression visualization and integration platform
FLA: Flag leaf area
FLL: Flag leaf length
FLW: Flag leaf width
GO: Gene ontology
GWAS: Genome-wide association study
KEGG: Kyoto encyclopedia of genes and genomes
KN: Kernel number per spike
LOD: The log of odds ratio
MAS: Marker-assisted selection
MQTL: Meta-QTL
MTAs: Marker trait associations
PH: Plant height
PVE: The explained phenotypic variation
QTL: Quantitative trait loci
RIL: Recombinant inbred line populations
*R*^*2*^: The proportion of phenotype variation value
SL: Spike length
SNP: Single nucleotide polymorphism
SSR: Simple sequence repeat
TGW: Thousand-grain weight
TPM: Transcripts per million
