# Supplementary material for: Deciphering key genomic regions controlling flag leaf size in wheat via integration of meta-QTL and in silico transcriptome assessment
Source: BMC Genomics. 2023 Jan 19;24:33. doi: 10.1186/s12864-023-09119-5 (PMC9854125; doi:10.1186/s12864-023-09119-5)
Supplement: Supplementary file 2 — Additional file 2: Figure S1. Frequency of candidate genes in each of 16 different proteins associated with flag leaf traits. [file 12864_2023_9119_MOESM2_ESM.docx]

**Additional file 2**

**Figure S1.**  Frequency of candidate genes in each of 16 different proteins associated with flag leaf traits.

**
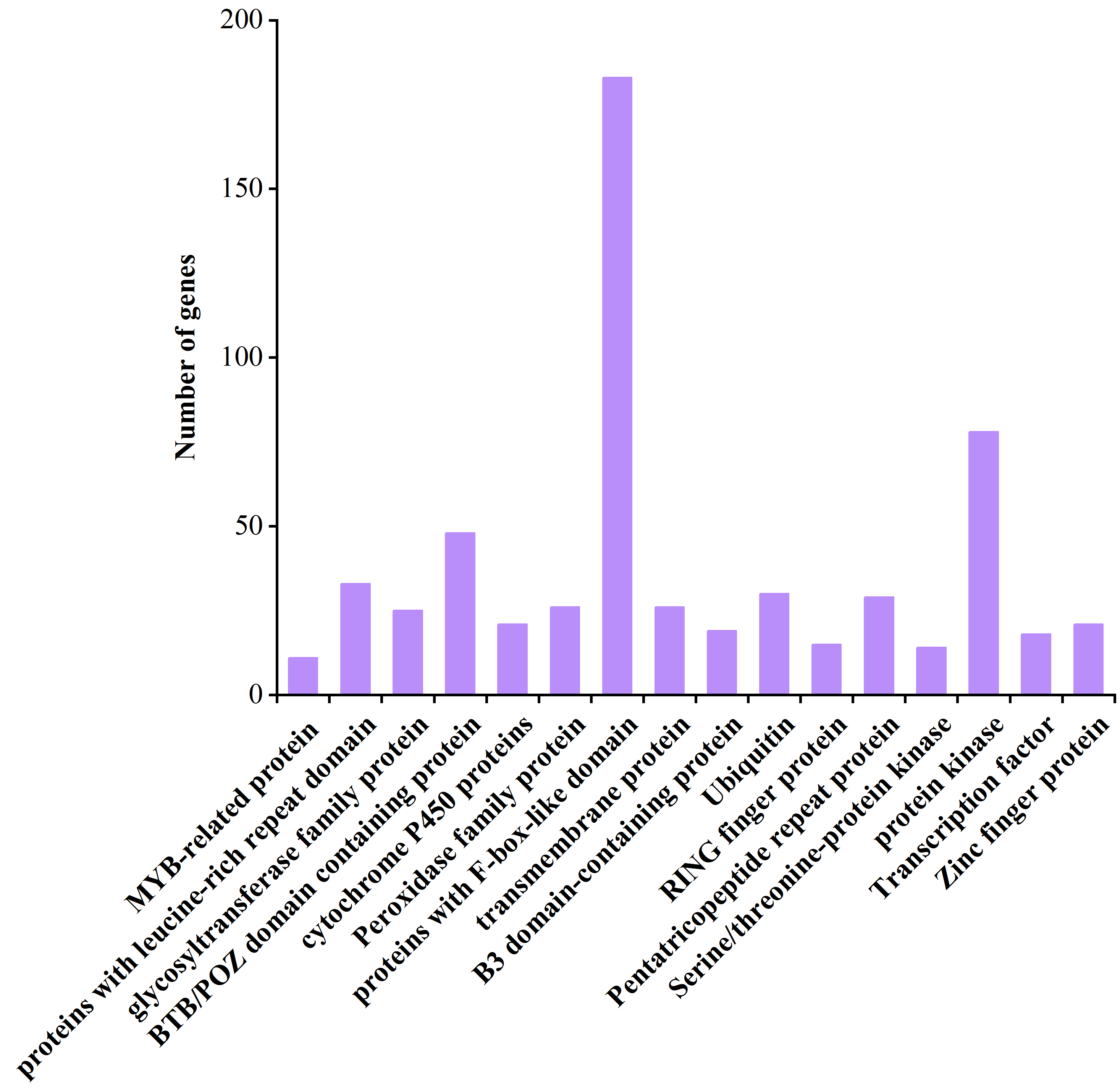
**
